# The Role of CD200 and CD43 Expression in Differential Diagnosis between Chronic Lymphocytic Leukemia and Mantle Cell Lymphoma

**DOI:** 10.4274/tjh.2017.0085

**Published:** 2018-05-25

**Authors:** Mesude Falay, Berna Afacan Öztürk, Kürşad Güneş, Yasin Kalpakçı, Simten Dağdaş, Funda Ceran, Gülsüm Özet

**Affiliations:** 1 University Ministry of Health, Ankara Numune Training and Research Hospital, Clinic of Hematology, Ankara, Turkey

**Keywords:** Chronic lymphocytic leukemia, Mantle cell lymphoma, Immunophenotyping, CD200, CD43

## Abstract

**Objective:**

Atypical chronic lymphocytic leukemia (CLL) is most frequently confused with mantle cell lymphoma (MCL). Several markers may contribute to the diagnosis of CLL. However, there is no consensus on which markers are needed to be used in flow cytometry for the diagnosis of CLL. The aim of the present study was to investigate the role of CD43 and CD200 markers in the differential diagnosis between CLL and MCL.

**Materials and Methods:**

To address this issue, 339 consecutive patients with CLL and MCL were included in the flow cytometry lymphoproliferative disease panel for evaluation of CD43 and CD200 expressions, but not in the Matutes scoring system.

**Results:**

CD200 was expressed in 97.3% of atypical CLL cases, whereas it was dimly expressed in only 6.1% of MCL cases. CD43 expression was 95.7% in atypical CLL cases. In the MCL cases, its expression rate was 39.4%.

**Conclusion:**

CD43 and CD200 were found to be more valuable markers than CD22, CD79b, and FMC7. CD43 and CD200 could also be considered as definitive markers in atypical CLL patients, for whom the Matutes scoring system remains ineffective.

## Abstract (tr)

**Amaç:**

İmmünfenotip olarak atipik kronik lenfositik lösemi (KLL) ile mantle cell lenfoma (MCL) sıklıkla karışabilmektedir. KLL tanısı için birçok marker kullanılmaktadır, ancak akım sitometride KLL tanısı için tam bir konsensüs oluşmamıştır. Bu çalışmada KLL ve MCL ayırıcı tanısında CD43 ve CD200 ifadeleri araştırılmıştır.

**Gereç ve Yöntemler:**

Matutes skorlama sisteminde olmayan CD43 ve CD200’ü akım sitometri lenfoproliferatif hastalık paneline dahil ederek 339 KLL ve MCL olgusunda incelenmiştir.

**Bulgular:**

Atipik KLL olgularının %97,3’ünde CD200 pozitifken MCL olgularının ise sadece %6,1’inde düşük oranda ifade ediliyordu. CD43’te atipik KLL olgularının %95,7’sinde ifade edilirken MCL olgularının %39,4’ünde donuk ifade ediliyordu.

**Sonuç:**

CD43 ve CD200; CD22, CD79b ve FMC7’ye göre daha anlamlı bulundu. CD43 ve CD200 Matutes skorlama sistemi skorunun yetersiz kaldığı KLL olgularının tanısında tamamlayıcı marker olarak kullanılabilir.

## Introduction

The World Health Organization (WHO) classification of hematolymphoid system neoplasms is based on clinical, morphological, immunophenotypic, and genetic features. Mature B-cell lymphoproliferative diseases (LPDs) account for more than 80% of hematolymphoid neoplasms [1]. Chronic lymphocytic leukemia (CLL) is the most frequent type of LPD [1,2]. Genetics has no role in the diagnosis of CLL, although there are numerous genetic abnormalities. The presence of persistent clonal B lymphocytosis (>5x10^9^/L lymphocytes) for more than 3 months is needed to make a diagnosis of CLL. It has characteristic morphological features, as well as immunophenotypic features in flow cytometry [1,2,3,4]. These include CD5+CD19+, CD23+, weak surface membrane immunoglobulins (sIg), and absent or low expression of CD79b and FMC7 [3,4]. Immunophenotyping has a major role in the diagnosis of CLL. However, CLL is a quite heterogeneous disease; for this reason, it can be difficult to diagnose [3,4,5,6,7]. Accordingly, a scoring system for the diagnosis of CLL was first defined in 1994 by Matutes et al. [8]. This scoring system consists of five parameters: CD5, CD22, CD23, FMC7, and sIg. In 1997, Moreau et al. [9] replaced CD22 by CD79b in the scoring system. According to this scoring system, a score of 4-5 indicates typical CLL and a score of 3 indicates atypical CLL, whereas a score of 0-2 excludes CLL [8,9]. Atypical CLL is most frequently confused with mantle cell lymphoma (MCL), which co-expresses CD5 and CD19 similarly to CLL [4,10,11,12,13,14,15,16]. Generally, MCL is more aggressive and requires a different therapeutic approach; therefore, differential diagnosis between these two diseases should be performed precisely. Histochemical or molecular tests [cyclin D1, SOX11, t(11;14)] can be used for differential diagnosis [4,12]. Molecular tests are not easily available, and they are time-consuming and more expensive. For this reason, reliable additional new markers have been investigated in cases in which the Matutes score is inadequate. Several markers such as CD200 and CD43 may contribute to the diagnosis of CLL. However, there is no consensus on which markers are needed to be used in flow cytometry for the diagnosis of CLL. In the present study, we aimed to investigate the role of markers that were included in our LPD panel in flow cytometry but not in the Matutes scoring system in the differential diagnosis between CLL and MCL.

## Materials and Methods

### Patients and Samples

The present study retrospectively evaluated the medical records of 339 patients diagnosed with CLL (n=306) and MCL (n=33) according to the WHO criteria [1]. For all patients, data on complete blood count and peripheral blood (PB) and/or bone marrow (BM) smear performed for morphological assessments were obtained. All atypical CLL patients were evaluated for cyclin D1 and/or t(11;14). Diagnosis of MCL was confirmed by immunohistochemical detection of cyclin D1 in BM biopsies or detection of t(11;14) by fluorescence in situ hybridization. SOX11 expression was not evaluated.

### Flow Cytometry Immunophenotyping

For flow cytometric study, fresh PB/BM samples were drawn into 4-mL K3-EDTA tubes (BD Vacutainer, USA) and studied immediately. Cells in suspension (2x10^6^ cells in 50-100 µL per tube) from the PB and BM samples were stained with monoclonal antibodies (MoAbs) directed against cell surface markers via a stain-lyse-and-then-wash direct immunofluorescence method [17]. The MoAbs used for labeling in flow cytometry were obtained from Beckman Coulter (BC, USA). A five-color staining was applied for all samples using the following fluorochrome-conjugated antibodies. MoAbs including fluorescein isothiocyanate (FITC), phycoerythrin (PE), phycoerythrin-Texas red (ECD), phycoerythrin cyanine 5 (PC5), and allophycocyanin (APC) were used for all patients: CD45/CD5/CD10/CD19/CD23, CD19/CD103/CD22/CD11c/CD25, CD5/CD20/sIgκ/sIgλ CD45, CD19/CD3/CD79b/CD22, and CD19/CD43/CD200/CD38. A tube containing Ig isotype controls for FITC/PE/ECD/PC5/APC was used for all patients. Data were immediately obtained at the end of sample staining using a flow cytometer (Navios, BC, USA) and Kaluza Flow Cytometry Analysis Software (BC, USA). For each sample, data from at least 10x10^4^ events per tube were obtained. Instrument alignment was confirmed daily using an alignment control bead (Flow-Check, BC, USA). The accuracy and precision of cell counts were tested using international quality controls purchased from the United Kingdom National External Quality Assessment Scheme (UK NEQAS LI, Sheffield, UK) (z-score range of -2.0 to 2.0). Briefly, CD19+ B cells were selected (at least 2000 events according to the threshold of the isotype control) from the data file using conventional gating strategies (forward and side scatter and the pattern of CD19 expression). As recommended by the British Committee for Standards in Haematology guideline [2], a cut-off value of 30% of lymphoid cells was accepted to indicate a positive result with a given antibody using the Kaluza software. The Matutes scoring was defined as ≥30% cell surface expression. In all patients, the same fluorescent-labeled MoAbs were used to ensure that the Matutes scoring was accurate. Diagnosis of LPD was established according to the WHO classification based on clinical data and morphologic, immunophenotypic, and genetic criteria. The revised Matutes scoring system [9], based on the immunophenotypic analysis of five membrane markers (CD5, CD23, FMC7, sIg, CD79b), was used to classify all patients. This scoring system assigns 1 point each for expression of CD5, CD23, and sIg and for lack of expression of CD79b and FMC7. A score of ≥4 indicates typical CLL patients and a score of 3 or a lack of CD23 indicates atypical CLL patients. In all patients, cyclin D1 and/or t(11;14) was used for the differential diagnosis. Diagnosis of MCL was confirmed by cyclin D1 and/or t(11;14).

### Statistical Analysis

Data analysis was performed using SPSS 15 for Windows (SPSS Inc., Chicago, IL, USA). Descriptive statistics were expressed as numbers and percentages. Categorical data were analyzed by multivariate forward stepwise regression analysis, Pearson’s chi-square test, or Fisher’s exact test as appropriate. A p-value of less than 0.05 was considered statistically significant.

## Results

The evaluation of 339 patients (100 females, 239 males) with mean age of 68±10.4 years (range: 31-87 years) revealed that median PB lymphocyte count at diagnosis was 19.8x10^9^ lymphocytes/L (range: 0.8-274x10^9^ lymphocytes/L). Of the patients, 306 (90.26%) had CLL and 33 (9.74%) had MCL (Table 1). According to the Matutes scoring of CLL patients, 121 (40%) patients had a score of ≥4 (of whom 105 (34.3%) had a score of 4 and 16 (5.2%) had a score of 5), 178 (58.2%) patients had a score of 3, 6 (2%) patients had a score of 2, and 1 patient (0.3%) had a score of 1 (Table 2). There was no significant difference between the typical and atypical CLL patients in terms of morphological evaluation. In all atypical CLL cases, cyclin D1 and/or t(11;14) were negative. The Matutes scores of the MCL patients with positive cyclin D1 and/or t(11;14) were 3 in 7 (21.2%) patients, 2 in 11 (33.3%) patients, and 1 in 15 (45.5%) patients. There were no MCL patients with a score of ≥4. Regarding CD22, CD79b, FMC7, and CD23 expressions in the Matutes score, CD23 expression was negative in 11 (3.5%) CLL patients (3 had typical CLL and 8 had atypical CLL), whereas it was positive in 6 (21.2%) MCL patients CD23 expression was not diagnostic for CLL but it was significantly more expressed in CLL patients (p<0.001). CD22, CD79b, and FMC7 expressions were highly positive in atypical CLL patients (96.2%, 81.6%, and 97.3%, respectively) (Table 3); however, the difference was not significant in the differential diagnosis between CLL and MCL (p=1.000, p=0.431, and p=1.000, respectively). CD79b expression was also positive in 38.8% of the CLL patients. No significant difference was found between the CLL and MCL patients regarding sIg expression intensity (p=0.385). Evaluations of CD38, CD43, and CD200expressions were included in the LPD panel but not in the Matutes scoring system (Table 2). While CD38 expression was moderate to strong in 93.9% of the MCL patients, it was dimly expressed in 24% of both atypical and typical CLL patients (p<0.001). When CD43 expression was evaluated, 95.7% of the patients with atypical CLL and 98.3% of the patients with typical CLL had moderate to strong expression. Among the MCL patients, CD43 expression was dim to moderate in 39.4% (p<0.001). When CD200 expression was evaluated, it was moderate to strong in 95.8% of the CLL patients (3.6% had negative expression), whereas it was dimly expressed only in 2 MCL patients (6.1%) (p<0.001; Table 2). There was no significant difference in CD200 expressions between the atypical and typical CLL patients. In the differential diagnosis of MCL and atypical CLL patients, multivariate forward stepwise regression analysis revealed the most determinant marker to be CD200 (p<0.001, 95% CI; Table 4).

## Discussion

The diagnosis of CLL is easy in the presence of characteristic immunophenotypic features (CD5+CD19+ dual-positive, CD23+, CD22-/low, CD79b-/low, sIg low, FMC7-, and CD20 low). However, it is difficult to make a differential diagnosis of CLL from MCL when immunophenotypic features are not typical. In the present study, CD43 and CD200 expressions, which were included in the LPD panel but not in the Matutes scoring system, were found significant in the differential diagnosis between CLL and MCL.

Immunophenotyping by flow cytometry, which is a frequently used method, is beneficial in the distinction of CLL from MCL [3,4,15]. However, there may be a problem for atypical immunophenotypes in which the Matutes score is ≤3. Therefore, it may be particularly difficult to distinguish some MCL cases from atypical CLL cases. CD23 positivity is the most characteristic feature of CLL [10,11]. Earlier studies have reported that CD23 negativity is a reliable marker in the distinction between CLL and MCL [15]. In the present study, while 2.1% of the typical CLL patients were CD23-negative, 21.2% of the MCL patients with positive t(11;14) were CD23-positive. However, according to our findings, CD23 alone was not efficient to make a differential diagnosis between CLL and MCL [12,13,16]. On the other hand, FMC7, which is an epitope of CD20, was expressed in 42.1% of the typical CLL patients and 97.3% of the atypical CLL patients. Similarly, the level of CD22 expression was closely correlated with CD20. CD79b expression was also positive in 38.8% of the CLL patients, which was considered in normal ranges. The percent positivity and intensity of CD79b expression in MCL, atypical CLL, and typical CLL is still controversial. CD22 and FMC7 expressions are generally higher in MCL patients, whereas in the present study, they were higher in both the CLL and MCL patients. For this reason, the majority of the patients (58.2%) were classified as having atypical CLL when Matutes scoring was used. Earlier studies stated that FMC7, CD79b, and CD22 are not efficient in making a differential diagnosis [1,17,18,19]. Every manufacturer produces MoAbs in different clones and different stains. There is a need for validation and standardization studies on these MoAbs. At this point, the present study had a limitation because the results were not checked with the use of different MoAbs of different clones from different manufacturers.

With regard to CD38, CD43, and CD200, which were not included in the Matutes scoring system, the different results obtained in the present study between the CLL and MCL patients could be partially explained by the individual differences among the patients as well as the absence of specific techniques and procedures in the flow cytometry. In the present study, CD38 expression was higher in the MCL patients than in the CLL patients (p<0.001) but heterogeneous in the CLL patients; thus, it was difficult to standardize. In addition, the LPD may have a fluctuating course [20,21,22,23]. All of these factors need to be taken into account while making a differential diagnosis between CLL and MCL.

CD43 expression was first defined in 1999 by Harris et al. [24] for the classification of malignant lymphomas. In the present study, CD43 expression was higher in the CLL patients compared with that in the MCL patients and it was quite effective in accurate classification of the patients having Matutes scores of ≤3 according to the classical classification (p<0.001) [25,26,27].

In the present study, while 95.8% of the CLL patients showed moderate to strong CD200 expression (3.6% had negative expression), 6.1% of the MCL patients showed positive CD200 expression (p<0.001). There was no significant difference in CD200 expressions between the atypical and typical CLL patients. Moreover, CD200 was constantly expressed in the typical CLL patients and was an excellent marker for its differential diagnosis from MCL, as previously shown in other studies [14,15,16,17,18,19].

### Study Limitation

The limitation of the present study was to not evaluate CD200 and CD43 expressions in other LPD groups. If these expressions were evaluated in other LPD groups, other diseases besides CLL and MCL would have also been evaluated with regard to CD200 and CD43 expressions. However, as the number of patients with other diseases was low in the present study, they were not included.

## Conclusion

In conclusion, there has not been a single marker identified yet to make a definite diagnosis of CLL by flow cytometry. Therefore, new markers for the differential diagnosis of CLL are under investigation. The results of the present study revealed that CD43 and CD200 in particular were more valuable markers than CD22, CD79b, and FMC7, which are within the scope of the Matutes scoring system. CD43 and CD200 could also be considered as definitive markers in atypical CLL patients for whom the Matutes scoring system remains ineffective. However, as with the other markers, their heterogeneous distribution and different rates of expression may still be in question. For this reason, large-scale harmonization studies are needed for patients with various diseases by defining standardized sample preparation and staining, as well as specific techniques. Identification of a new scoring system following these studies would also be beneficial.

## Figures and Tables

**Table 1:**
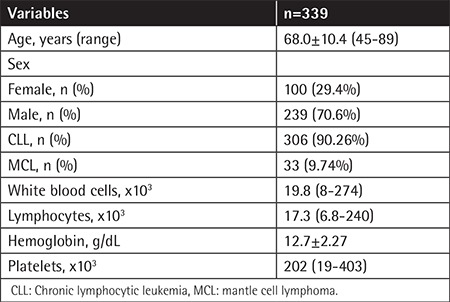
Demographic features.

**Table 2:**
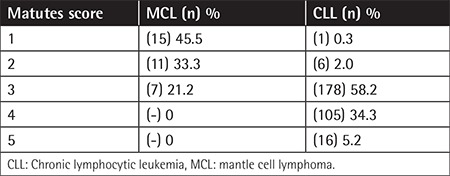
Mantle cell lymphoma and chronic lymphocytic leukemia patients’ Matutes scores.

**Table 3:**
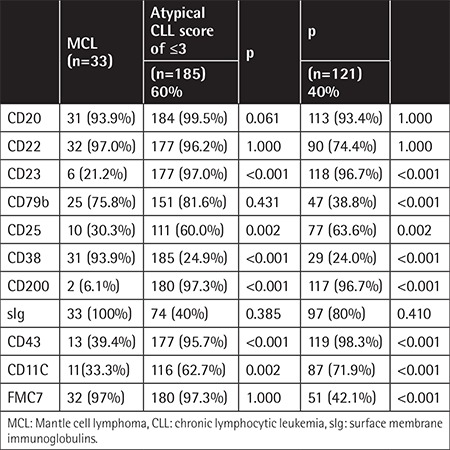
Distribution of cases by marker positivity in the differential diagnosis of mantle cell lymphoma and atypical chronic lymphocytic leukemia score of ≤3, chronic lymphocytic leukemia score of ≥4.

**Table 4:**
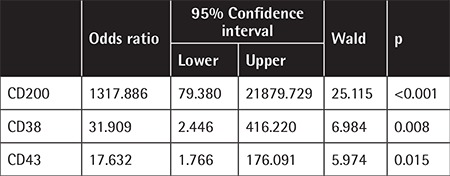
Multivariate analysis for mantle cell lymphoma and atypical chronic lymphocytic leukemia discrimination.

## References

[1] Campo E, Swerdlow SH, Harris NL, Pileri S, Stein H, Jaffe ES (2011). The 2008 WHO classification of lymphoid neoplasms and beyond: evolving concepts and practical applications. Blood.

[2] Oscier D, Dearden C, Eren E, Fegan C, Follows G, Hillmen P, Illidge T, Matutes E, Milligan DW, Pettitt A, Schuh A, Wimperis J, British Committee for Standards in Haematology (2012). Guidelines on the diagnosis, investigation and management of chronic lymphocytic leukaemia. Br J Haematol.

[3] Costa ES, Pedreira CE, Barrena S, Lecrevisse Q, Flores J, Quijano S, Almeida J, del Carmen García-Macias M, Bottcher S, Van Dongen JJ, Orfao A (2010). Automated pattern-guided principal component analysis vs expert-based immunophenotypic classification of B-cell chronic lymphoproliferative disorders: a step forward in the standardization of clinical immunophenotyping. Leukemia.

[4] Braylan RC (2004). Impact of flow cytometry on the diagnosis and characterization of lymphomas, chronic lymphoproliferative disorders and plasma cell neoplasias. Cytometry A.

[5] Dreyling M, Hiddemann W, European MCL Network (2009). Current treatment standards and emerging strategies in mantle cell lymphoma. Hematology Am Soc Hematol Educ Program.

[6] Sánchez ML, Almeida J, Vidriales B, López-Berges MC, García-Marcos MA, Moro MJ, Corrales A, Calmuntia MJ, San Miguel JF, Orfao A (2002). Incidence of phenotypic aberrations in a series of 467 patients with B chronic lymphoproliferative disorders: basis for the design of specific four-color stainings to be used for minimal residual disease investigation. Leukemia.

[7] Dronca RS, Jevremovic D, Hanson CA, Rabe KG, Shanafelt TD, Morice WG, Call TG, Kay NE, Collins CS, Schwager SM, Slager SL, Zent CS (2010). CD5-positive chronic B-cell lymphoproliferative disorders: diagnosis and prognosis of a heterogeneous disease entity. Cytometry B Clin Cytom.

[8] Matutes E, Owusu-Ankomah K, Morilla R, Garcia Marco J, Houlihan A, Que TH, Catovsky D (1994). The immunological profile of B-cell disorders and proposal of a scoring system for the diagnosis of CLL. Leukemia.

[9] Moreau EJ, Matutes E, A’Hern RP, Morilla AM, Morilla RM, Owusu-Ankomah KA, Seon BK, Catovsky D (1997). Improvement of the chronic lymphocytic leukemia scoring system with the monoclonal antibody SN8 (CD79b). Am J Clin Pathol.

[10] Medd PG, Clark N, Leyden K, Turner S, Strefford JA, Butler C, Collins GP, Roberts DJ, Atoyebi W, Hatton CS (2011). A novel scoring system combining expression of CD23, CD20, and CD38 with platelet count predicts for the presence of the t(11;14) translocation of mantle cell lymphoma. Cytometry B Clin Cytom.

[11] Kroft SH (2008). Uncovering clinically relevant phenotypic variations in malignancies: CD23 in mantle cell lymphoma. Am J Clin Pathol.

[12] Schlette E, Fu K, Medeiros LJ (2003). CD23 expression in mantle cell lymphoma: clinicopathologic features of 18 cases. Am J Clin Pathol.

[13] Gao J, Peterson L, Nelson B, Goolsby C, Chen YH (2009). Immunophenotypic variations in mantle cell lymphoma. Am J Clin Pathol.

[14] Kilo MN, Dorfman DM (1996). The utility of flow cytometric immunophenotypic analysis in the distinction of small lymphocytic lymphoma/chronic lymphocytic leukemia from mantle cell lymphoma. Am J Clin Pathol.

[15] Kaleem Z (2006). Flow cytometric analysis of lymphomas: current status and usefulness. Arch Pathol Lab Med.

[16] Gong JZ, Lagoo AS, Peters D, Horvatinovich J, Benz P, Buckley PJ (2001). Value of CD23 determination by flow cytometry in differentiating mantle cell lymphoma from chronic lymphocytic leukemia/small lymphocytic lymphoma. Am J Clin Pathol.

[17] Stewart CC, Stewart SJ (2001). Immunophenotyping. Curr Protoc Cytom.

[18] Kraus TS, Sillings CN, Saxe DF, Li S, Jaye DL (2010). The role of CD11c expression in the diagnosis of mantle cell lymphoma. Am J Clin Pathol.

[19] Angelopoulou MK, Kontopidou FN, Pangalis GA (1999). Adhesion molecules in B-chronic lymphoproliferative disorders. Semin Hematol.

[20] Hamblin TJ, Orchard JA, Ibbotson RE, Davis Z, Thomas PW, Stevenson FK, Oscier DG (2002). CD38 expression and immunoglobulin variable region mutations are independent prognostic variables in chronic lymphocytic leukemia, but CD38 expression may vary during the course of the disease. Blood.

[21] Matrai Z (2005). CD38 as a prognostic marker in CLL. Hematology.

[22] Kröber A, Seiler T, Benner A, Bullinger L, Brückle E, Lichter P, Döhner H, Stilgenbauer S (2002). V(H) mutation status, CD38 expression level, genomic aberrations, and survival in chronic lymphocytic leukemia. Blood.

[23] Thompson PA, Tam CS (2014). CD38 expression in CLL: a dynamic marker of prognosis. Leuk Lymphoma.

[24] Harris NL, Jaffe ES, Diebold J, Flandrin G, Muller-Hermelink HK, Vardiman J, Lister TA, Bloomfield CD (1999). World Health Organization classification of neoplastic diseases of the hematopoietic and lymphoid tissues: report of the Clinical Advisory Committee meeting-Airlie House, Virginia, November 1997. J Clin Oncol.

[25] Durrieu F, Geneviève F, Arnoulet C, Brumpt C, Capiod JC, Degenne M, Feuillard J, Garand R, Kara-Terki A, Kulhein E, Maynadié M, Ochoa-Noguera ME, Plesa A, Roussel M, Eghbali H, Truchan-Graczyk M, de Carvalho Bittencourt M, Feugier P, Béné MC (2011). Normal levels of peripheral CD19+CD5+ CLL-like cells: toward a defined threshold for CLL follow-up-a GEIL-GOELAMS study. Cytometry B Clin Cytom.

[26] Deneys V, Michaux L, Leveugle P, Mazzon AM, Gillis E, Ferrant A, Scheiff JM, De Bruyère M (2001). Atypical lymphocytic leukemia and mantle cell lymphoma immunologically very close: flow cytometric distinction by the use of CD20 and CD54 expression. Leukemia.

[27] Jung G, Eisenmann JC, Thiébault S, Hénon P (2003). Cell surface CD43 determination improves diagnostic precision in late B-cell diseases. Br J Haematol.

